# Mind the gap between policy imperatives and service provision: a qualitative study of the process of respiratory service development in England and Wales

**DOI:** 10.1186/1472-6963-8-248

**Published:** 2008-12-04

**Authors:** Sonya Hamilton, Guro Huby, Alison Tierney, Alison Powell, Tara Kielmann, Aziz Sheikh, Hilary Pinnock

**Affiliations:** 1 Allergy & Respiratory Research Group, Centre of Population Health Sciences: GP Section, University of Edinburgh, 20 West Richmond St, Edinburgh, EH8 9DX, UK; 2 Centre for Integrated Healthcare Research, School of Health in Social Science, University of Edinburgh, Teviot Place, Edinburgh, EH8 9AG, UK; 3 Department of Social Medicine, University of Bristol, Canynge Hall, 39 Whatley Road, Bristol, BS8 2PS, UK

## Abstract

**Background:**

Healthcare systems globally are reconfiguring to address the needs of people with long-term conditions such as respiratory disease. Primary Care Organisations (PCOs) in England and Wales are charged with the task of developing cost-effective patient-centred local models of care. We aimed to investigate how PCOs in England and Wales are reconfiguring their workforce to develop respiratory services, and the background factors influencing service redesign.

**Methods:**

Semi-structured qualitative telephone interviews with the person(s) responsible for driving respiratory service reconfiguration in a purposive sample of 30 PCOs. Interviews were recorded, transcribed, coded and thematically analysed.

**Results:**

We interviewed representatives of 30 PCOs with diverse demographic profiles planning a range of models of care. Although the primary driver was consistently identified as the need to respond to a central policy to shift the delivery of care for people with long-term conditions into the community whilst achieving financial balance, the design and implementation of services were subject to a broad range of local, and at times serendipitous, influences. The focus was almost exclusively on the complex needs of patients at the top of the long-term conditions (LTC) pyramid, with the aim of reducing admissions. Whilst some PCOs seemed able to develop innovative care despite uncertainty and financial restrictions, most highlighted many barriers to progress, describing initiatives suddenly shelved for lack of money, progress impeded by reluctant clinicians, plans thwarted by conflicting policies and a PCO workforce demoralised by job insecurity.

**Conclusion:**

For many of our interviewees there was a large gap between central policy rhetoric driving workforce change, and the practical reality of implementing change within PCOs when faced with the challenges of limited resources, diverse professional attitudes and an uncertain organisational context. Research should concentrate on understanding these complex dynamics in order to inform the policymakers, commissioners, health service managers and professionals.

## Background

The increasing prevalence of long-term conditions is acknowledged as an important challenge for healthcare services globally. [1,2] The need to care for those with long-term disease in an ageing population places considerable demands on existing health and social care resources.

Respiratory conditions, currently responsible for 7% of deaths in the UK,[1] are predicted to become one of the leading five causes of chronic ill health globally by 2020. [3] Chronic Obstructive Pulmonary Disease (COPD) is responsible for one in eight emergency admissions to hospital,[4,5] Following two high-profile reports which highlighted the need for personalised, structured and integrated care for people with COPD in order to manage the disease burden more effectively,[5,6] a National Service Framework (NSF) has been commissioned.

**Figure 1:**
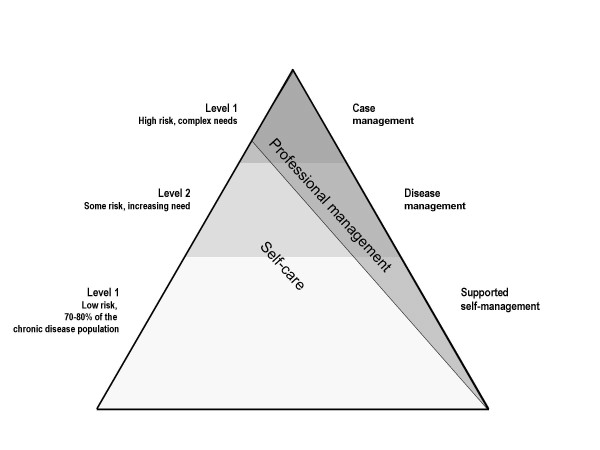
**Pyramid of care for long-term conditions**. (adapted from *Improving Chronic Disease Management*. [2]).

In the UK, a number of policies have been introduced to address the challenge of caring for people with long-term conditions. Learning from US managed care programmes, the long-term condition pyramid (LTC pyramid) is suggested as an important framework for designing services,[7] with community matrons providing case management for people with complex needs at the top of the pyramid (see figure 1). The Quality and Outcomes Framework of the General Medical Services contract aims to improve primary care standards, [8] and investment in Expert Patient programmes and health literacy support self-care at lower levels of the pyramid. [9]

'Care Closer to Home' is widely promoted as offering a cost-effective alternative to expensive hospital treatment, with specific initiatives such as Hospital at Home schemes, and GPs with special interests (GPwSIs), seen as important components of intermediate care services integrating primary and secondary care. [2,5,7,10] In England and Wales, PCOs are charged with the responsibility to commission services to implement these policies according to local need. Although reviews of the evidence on diffusion of innovation in the health service, [11] and summaries of advice on achieving organisational change in the NHS are available, [12] there is a need to understand how policy is implemented in practice amidst current changes and reorganisations within the NHS.

Our study aimed to investigate how PCOs (i.e. Primary Care Trusts in England and Local Health Boards in Wales; freestanding statutory NHS bodies with responsibility for delivering healthcare and health improvements to their local areas) reconfigure their workforce to develop respiratory services and to meet the needs of people with long-term conditions. Our previous work suggested that up to a third of PCOs were considering including GPwSIs in their respiratory service,[13] based on evidence that they can safely provide care for a proportion of patients otherwise referred to secondary care,[14] and that clinical outcomes are similar, with patients often equally or more satisfied with the service. [15-17] Our study, therefore specifically aimed to study the development of a GPwSI-centred service models within the context of other (often nurse-led) models.

We here report the first phase of the study in which we explored the context, drivers, barriers and facilitators to respiratory service reconfiguration in a purposefully selected sample of PCOs in England and Wales, representing a broad spectrum of attitudes and levels of development in the reconfiguration of respiratory services. This 'baseline' phase had the dual objective of enabling us to select four PCOs for in-depth case study (to be reported in due course) and also of providing the broad context for further evaluation.

## Methods

This study was undertaken with the ethics approval of the Southeast Multi-Centre Research Ethics Committee and governance approval from all participating PCOs. [18] All participants provided informed consent.

We recruited a purposeful sample of PCOs, representing a broad spectrum of potentially relevant factors and influences, including demographic and geographic profile, existing or planned models of community-based respiratory care. As the primary interest of our study was the role of GPwSIs, we specifically sought a number of PCOs with GP or GPwSI involvement in reconfiguring respiratory services. Our initial selection was based on our knowledge of PCOs' intentions from a previous survey,[13] and on expressions of interest received in responses to the publication of the General Practice Airways Group *Respiratory GPwSI resource pack*. [19] These were supplemented by snowball sampling to identify PCOs reputed to have in place or be planning novel models of care.

At the time of the interviews there was a total of 330 PCOs, however we were aware of imminent mergers, which subsequently reduced the number of PCOs to 110. We took this into consideration, when recruiting in order, for example, to avoid overlap where PCOs were already working closely with their prospective partners.

We approached PCOs by letter, followed up by a phone call, requesting a 45 minute telephone interview with the person(s) responsible for driving the reconfiguration of respiratory services or, in the case of PCOs not planning reconfiguration of respiratory services, the person responsible for other comparable chronic disease services in the PCO. We planned to recruit until we identified no new models of care and were satisfied we had reached data saturation.

Based on our previous work, [13,20] and our understanding of current policies and discussions relating to the management of long-term conditions, [2,5-7,10,21] we devised a semi-structured interview schedule, collecting data on size and demographics of the PCO, financial and organisational context, the current priorities, preferred model of care for respiratory disease, key drivers, barriers and facilitators (see Additional file 2, Appendix 1 for the full schedule). The topic guide was reviewed by the multi-disciplinary team in an iterative process as the interviews progressed.

The interviews were conducted by one researcher (AT) who made extensive field notes on pre-structured forms. Interviews were audio-recorded (apart from interviews 1 and 2 because of technical problems) and fully transcribed. Analysis of the interview data was undertaken by two researchers (SH and HP) using the thematic method described by Zeibland *et al*. [22] Emergent themes were discussed by all members of the multidisciplinary team during project meetings and workshops.

## Results

### Participants

We sent a postal invitation to 110 PCOs between February and June 2006; 40 agreed to consider our request. After gaining permission from line managers, 30 identified a suitable person for an interview. The demographic details, merger and financial status of the PCOs and the professional role of the interviewees are summarised in table 1 (see additional file 1).

### Models of care

Within the 30 sampled PCOs, we identified a range of respiratory service models, often including a combination of approaches, with multidisciplinary teams providing a respiratory service. We reached saturation in terms of the service models identified.

In summary, we have categorised these models according to the main focus of the model, as described by the interviewee's description.

• Nine PCOs specifically involved GPs, either as GPwSIs or as less formal arrangements with local 'interested GPs'

• Five were developing, or considering developing, respiratory GPwSI services.

• Sixteen had, or were developing, a role for community matrons in COPD care.

• Fifteen were nurse-led models, and a further seven included nurses in multi-disciplinary respiratory teams.

• Three were developing models incorporating consultants working in the community.

• Two PCOs were not prioritising respiratory care.

The models were in varying stages of development and implementation at the time of the interviews, but the fluidity of the process, and variability between different aspects of reconfiguration within individual PCOs made it impossible to give a meaningful indication of the phase of development.

**Figure 2:**
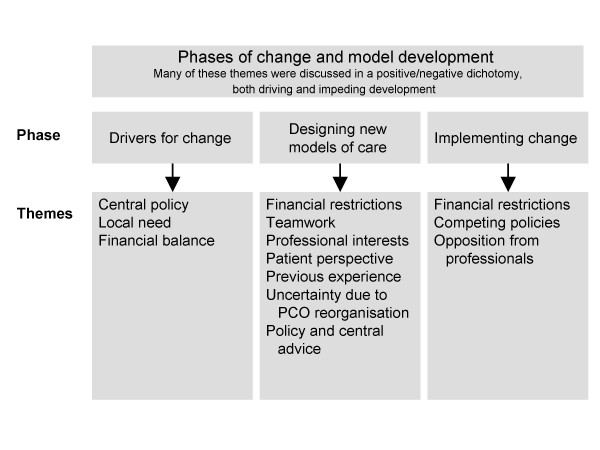
Summary of the phases of change and model development.

Throughout the interviews, the impact of change emerged as an important theme, which in many cases, was discussed in terms of a positive/negative dichotomy, both driving and impeding development. Reconfiguration of respiratory services was discussed within the context of the changing environment of the NHS in England and Wales, as at the time of the interviews, many of the Primary Care Organisations were merging, and/or undergoing structural reorganisation. Change impacted on all stages of respiratory service development from the initial drivers through the design phase to the implementation. We identified three phases of change and model development (summarised in figure 2): 1) Drivers for change, 2) Designing new models of care, and 3) Implementing change.

### I Drivers for change

#### Central policy

Many interviewees described the primary drivers to redevelopment as being central policies, particularly on shifting care into the community, the proactive management of long-term conditions and broadening of professional roles. The impending PCO mergers and commissioner-provider split provided a fluid and uncertain context for these changes.

*"..again I think PCO initiatives seem to be driven from central government which, you know, is understandable to a certain extent but the nature is that it tends to, unless you're very different and you're very enthusiastic you'll find that to implement any change is extremely difficult." *(PCO 14: GPwSI service, Interviewee: GPwSI)

*"...with the focus on cutting out-patients and particularly follow-ups there is a, the Trust has been put under, the consultants have been put under pressure themselves and so they're desperate for solutions. And so when we came along with some solutions, they were very keen to listen." *(PCO 6: Respiratory nurse service, Interviewee: Commissioner)

#### Local need

Recognition that change was needed to enhance local patient care was another important driver. Several PCOs were investing time and money in exploring local need with scoping exercises, or audits of service use, and a few were commissioning interviews and focus groups to help them understand the patients' perspective. Some PCOs valued the input of local practitioners as a means of gauging patient needs, though others were concerned that clinical perspectives might not always reflect those of patients.

*"Actually I think we have a very lively input from patients that we've made sure that *[the] *patient voice is at the centre of this. Our patients have said to us what is important for them and our service development group has made that a key priority. We've had some effort to encourage practices to take onboard a patient, a patient advisor." *(PCO 8, Respiratory nurse service, Interviewee: Service development manager)

*"...my own driver is really an interest in respiratory because I feel that as a group of patients over the years with the way that the primary care has gone certainly we've had NSF for coronary heart disease and diabetes, those who have respiratory problems have sort of been neglected to a sort of second division and I feel that that's particularly unfortunate given the huge amount of morbidity that's around with regards to respiratory disease..." *(PCO 14: GPwSI service, Interviewee: GPwSI)

#### Financial balance

The imperative to achieve financial balance was frequently cited as a driver for change. Budgetary and resource restrictions both drove service redesign by imposing a need for cost saving alternatives to hospital admissions, and acted as a major barrier as plans were shelved to save money.

*"Well the top priority, I am sure you are going to hear this everywhere, is financial, absolutely nothing to do with redesign, but that is the absolute top." *(PCO 3: GPwSI-led team, Interviewee: Commissioner)

*"...although current changes are said to be clinically led, the truth is they aren't. There's a significant gap between rhetoric and reality, which leaves clinicians exasperated, because their commitment to the well-being of their patients comes second to economic and political forces." *(PCO 14: GPwSI service, Interviewee: GPwSI)

### II Designing new models of care

#### Financial strategy

Almost all interviewees spoke of how financial restrictions impacted on the design of respiratory services. In some cases there was insufficient funding to develop a desired service: in others service development proceeded successfully only to have progress (often suddenly) aborted due to removal of funding. Models were often chosen because of their cost saving potential. In some cases these were not the preferred models, however financial restrictions did not allow for the more expensive (yet considered potentially better) model of care. Specifically, a GPwSI service was often rejected as being too expensive in relation to other options. Sometimes the choice of model was dictated by the presence of a funding stream for a specific model of care (for example: charity funding to start up an asthma education project for parents, pharmaceutical company sponsorship for pulmonary rehabilitation, or funding for initiatives to attract GPs to under-doctored areas used to support GPwSI training).

*"And there was some LDP *[Local Delivery Plan] *money which was put aside for chronic disease management which, fortunately for me, wasn't earmarked for any specific project and, so, what we did, we have a clinical reference group for respiratory diseases which covers all areas of the health economy and we put together a business plan basically which identified that we, we think we can reduce emergency admissions by 30% or more by implementing this team and the investment in the team is more than paid for by the reduction in admissions." *(PCO 6: Respiratory nurse service, Interviewee: Commissioner)

*".... and then we'll see what we can do about it. Either it will be through pots of money that people have got stashed away, it might be trust fund, it might be sponsorship etc. We haven't got quite there yet...money is always a barrier." *(PCO 11: Respiratory nurse service, Interviewee: Service development manager)

#### Teamwork

Teamwork was an enabler of change and service redesign in those PCOs who used the expertise available in primary and/or secondary care. Although managers and primary and secondary care clinicians could have different visions, alignment of perspectives could enable change.

*"...well I work, and am working at the moment closely with our lead consultant and our lead GP in [town] on modernising and developing alternative COPD services and I would like to say that I think that that has been instrumental in bringing about the kind of changes and changed service that we are now developing." *(PCO 3: GPwSI-led team, Interviewee: Commissioner)

*"I think the PCO driving force is the economic side of things... so I do feel that I'm basically trying to drive through a clinical area but obviously understand that you will only achieve these things if you satisfy other peoples aims as well " *(PCO 14: GPwSI service, Interviewee: GPwSI)

#### Professional interests

The presence of professional support or opposition was highlighted as an important factor influencing choice of model redesign. Some interviewees described how clinicians from primary or secondary care could actively "*champion" *preferred models or conversely how opposition (for example from consultants) could mean that certain choices were avoided. Examples were cited where the narrow perspective of a professional had restricted the possibilities of developing new ways of working, and PCOs had subsequently adopted strategies to counterbalance vested interests. More practically, availability of an individual with professional expertise and interest could determine whether a GPwSI or specialist nurse service was selected.

*"And fantastically the consultants, you know, they send me articles they see in Thorax about, I got sent one about GPwSIs the other day, and it's the first time that a consultant has actually come and said, 'Actually d'you know, there might be a role for a GPwSI somewhere in this'. I practically fell off my chair." *(PCO 6: Respiratory nurse service, Interviewee: Commissioner)

*" [The GPs] solution to service re-design was to go well we're going to have a GP with a special interest, that's the solution because, you know, that's the way I look at life, that obviously is the solution because GPs and primary care are the way forward." *(PCO 10: Respiratory nursing team, Interviewee: Nursing manager)

*"Nobody's come forward and expressed an explicit interest in becoming a GP with a special interest and so it hasn't featured in the model. However, the model is quite open to different ways of working so if that was to happen then it would fit nicely..." *[PCO 23: Respiratory nurse team. Interviewee: Nursing manager]

#### Patient perspective

Patient views on existing services and proposed redesign were actively sought by some PCOs, usually in the form of satisfaction surveys, though a few were commissioning interviews and focus groups to help them understand the patients' perspective. Local networks also provided opportunities to identify patient perspectives.

*"We're doing a full review of unscheduled care services at the moment so we're looking at all of that. And understanding what sort of deal patients get in an urgent or emergency situation, so that's being independently evaluated by [local university]." *(PCO 26. Community matrons. Interviewee: Nursing manager)

*"The inner city area has got a high Asian minority population but we have got an excellent public involvement manager involved in quite a numerous amount of projects within the inner city. And we've got good engagement from those minority ethnic groups.." *(PCO 22: respiratory nurse and consultant outreach service, Interviewees: Service development manager)

#### Previous experience

Decisions about models of care were influenced, both positively and negatively, by previous experience. This could be the personal experience of a person involved in redesign, or the PCO may have had success (or otherwise) with specific models in other long-term condition areas.

*"... having led on GPwSIs in orthopaedics and dermatology I personally understood the processes needed to put a GPwSI in place and therefore it didn't seem a huge problem to set it up, I felt as though I was quite familiar with what we needed to do." *(PCO 3: GPwSI-led team, Interviewee: Commissioner)

*"Yes, yes I mean we wouldn't rule out the GPwSI model but I think what we have found with experience from elsewhere about the cost of the GPwSI service actually sometimes they work out more expensive than appointing a consultant." *(PCO 19: Respiratory nurse service, Interviewee: Service development manager)

#### Uncertainty about PCO reorganisation

Many interviewees commented that the chaos and uncertainty associated with the imminent PCO reorganisation acted as a major block to effective development. Instability and lack of job security within PCOs due to the impending reorganisation meant that managerial positions remained vacant causing the planning process to stall. By contrast, however, several interviewees spoke positively of the potential for expanding their successful respiratory services to their future partner PCOs, or spoke optimistically of an opportunity to develop a new service.

*"I suppose it's not impossible that the reorganisation of the PCOs could be a great opportunity, in the sense that it's a new start with a new, newly formed organisation*. (PCO 7: Respiratory nursing service, Interviewee: Nursing manager)

*"...you just don't know and I think that degree of uncertainty is creating lots and lots of problems and it's hard to see where improvement is going to come when there is this environment of uncertainty which undoubtedly will last for quite a number of months once the mergers start, who's going to hire, who's going to fire, it may well mean that I would have to work with somebody else from another PCO who's totally disinterested in respiratory areas in which case we're really quite limited, so it is a worry but however we do our best to try and see if we can improve things." *(PCO 14: GPwSI service, Interviewee: GPwSI)

#### Policy and central advice

Many interviewees commented that specific policies and frameworks had a major influence on their thinking, citing 'the NHS Plan', 'Care Closer to Home' or 'LTC pyramid' in support of their plans for redesign, though interpreted these documents in the context of their local situation,

*"In terms of, the current work, our focus of effort has been the Kaiser Triangle*, [LTC pyramid]* has been looking at the top of the triangle for those most vulnerable patients. And putting locality systems in place, we don't have community matrons down here in [area], for several reasons. We had fairly well established intermediate care services and we felt that the community matrons would cause upset to our established intermediate care teams." *(PCO 27: up-skilling existing primary care, Interviewee: Service development manager)

### III Implementing change

Careful design and planning did not always ensure successful implementation. Policies, such as 'Payment by Results' (PbR), [23] could work against the service redesign, causing tension between the acute trust and the PCO. In some cases, service design proceeded successfully only to have a key appointment or initiative stopped (sometimes very suddenly) due to lack of funding.

*Interviewer: "Okay and do you encounter any obstacles or barriers to introducing these changes?" Interviewee 18: "Money." *(PCO 18: Respiratory nursing service, Interviewee: Commissioner)

*"I mean we had plans drawn up to fund myself and a respiratory nurse specialist in the community and this was going to be part of the local development plan but at the twelfth hour, the eleventh hour I should say, the PCO pulled the plug on it because they had no money, so I found out within sort of a week of this meant to have been going ahead that it wasn't going to go ahead, so we had no funds." *(PCO 14: GPwSI service, Interviewee: GPwSI)

Some interviewees described how implementation of the newly designed changes could be impeded by reluctant members of the healthcare teams, perhaps perceiving the proposed changes as a threat. There was particular emphasis on the need to change the medical culture for the new models to be accepted.

*"...so having set that up we are now looking at how we can develop it and take it a bit further but also just to get our GPs to make use of it is, you know... old habits die hard and they're used to referring to the hospital, you know, and we've got to try and turn them around. And the other big, big challenge is that the hospital consultants are very, very reluctant to send even follow up patients to our GPwSI and changing that culture is exceedingly difficult..." *(PCO 3: GPwSI-led team, Interviewee: Commissioner)

*"Well, we have had quite a lot of resistance from the respiratory team, I have to say, the manager, who will say to me, 'Oh yeah, it's a great idea'. But then the matrons struggle to get a service running with the respiratory nurse, because I think the respiratory nurse who's new is right in the middle of it all, between her manager and my matrons." *(PCO 7: Respiratory nursing service, Interviewee: Nursing manager)

## Discussion

Against a backdrop of uncertainty due the impending reorganisation and, in some cases, large financial deficits, the PCOs in our study sought to marshal their resources to develop new services to meet the increasing needs of a population with long-term respiratory conditions. Although the primary driver for this reconfiguration was consistently identified as the central policy to shift care for people with long-term conditions cost-effectively into the community, the design and implementation of new services was subject to a broad range of local and at times serendipitous influences which could, and often did, derail the process. Some interviewees described teams of clinicians and managers able to balance policy requirements and local needs in order to develop innovative care, albeit limited by financial restrictions and often with an uncertain future. Most, however, highlighted the many barriers to progress describing initiatives suddenly shelved for lack of money, progress impeded by reluctant clinicians, plans for reducing hospital care thwarted by 'Payment by Results' and a PCO workforce demoralised by the upheaval and job insecurity of a merger. For many of our interviewees, there was a large gap between policy rhetoric and practical reality.

### Limitations and strengths

Our participants may not have encompassed the full range of contexts in PCOs in England and Wales, however, we purposefully sampled trusts with a wide geographic and demographic spread and a range of proposed respiratory service models and in an attempt to minimise this risk we continued to recruit until saturation was reached. The 30 PCOs who agreed to participate may have been the most enthusiastic about reconfiguring services, however, our purposive sampling included one PCO with no intention to develop a respiratory service and several with very limited plans. In addition, the models described by the participants echoed those identified by a national survey. [13] Our data are derived from a single interview in each PCO, and although we standardised our requests to PCOs, asking to speak to the person responsible for driving the reconfiguration of respiratory services, some interviewees may not have been fully aware of the situation in their PCO. The interviewees had a range of clinical and/or managerial roles, and we recognise that their answers and perceptions will have reflected their individual perspectives. Interviewees may have omitted to mention some issues, though we used a structured topic guide to ensure that we asked specifically about relevant issues.

A major strength of the study is the multidisciplinary expertise (clinical, health service management, anthropological) available within the study team, ensuring balanced conclusions. We continued interviews until we reached saturation with regard to models of care.

### Interpretation of findings in relation to previously published work

Although the approach varied, almost all the developments described by our interviewees addressed the complex needs of patients at the top of the LTC pyramid, and focussed predominantly on reducing admissions. [2,7] Even if predictive models can accurately identify 'at risk' patients, [24] a narrow focus overlooks the importance of ensuring early diagnosis and strengthening disease management and supported self-care for those at lower levels of the pyramid to prevent progression and future escalation of care needs, [5,6,25] and perpetuates some of the limitations of the reactive approach to acute care. Short term planning (often no further than the end of the current financial year), limited resources and the uncertainty of imminent PCO reorganisation were amongst the factors identified by our participants as barriers to developing broader strategies.

Integration across primary and secondary care, and enabling collaboration between multidisciplinary teams of healthcare professionals, are enshrined in policy, [26-28] widely advocated in discussion, [5,29-32] and supported by some evidence. [33,34] The few PCOs in our study with multidisciplinary teams in place integrated between the acute sector and the community seemed better placed to address all levels of the LTC pyramid with their planned respiratory services, providing some support for the fundamental importance of multidisciplinary coordination of care in realising the potential for improved patient care. [34,35]

Our data identify a significant gap between aims and desires at the policy level, and how services are designed and implemented at ground level. Whilst policies were described as significant drivers of change, our interviewees discussed many other important factors impacting on practical service reconfiguration. The shape and effectiveness of service development are influenced by perceived local patient need, professional attitudes and workforce issues such as availability of potential GPwSIs. Development proceeds in an environment overshadowed by uncertainty and financial restrictions. The manner and success with which PCOs translate the aspirations of policy into reality appear to be very variable. As a result, services can look very different to users from PCO to PCO, potentially raising concerns about inequity. There is a need to understand why some trusts succeed in reconfiguring services despite the challenges whilst others flounder, in order to inform policymakers, commissioners, health service managers, professionals, and educationalists about effective strategies to implement policy. [36,37]

This paper is a descriptive piece providing broad, baseline context for further in-depth evaluation in subsequent phases of our study. The models we identified can be defined as innovations in health care: 'i.e. novel sets of behaviours, routines and ways of working, which are directed at improving health outcomes, administrative efficiency, cost-effectiveness or the user experience, and which are implemented by means of planned and co-ordinated action'. [38] Uptake and implementation of health innovations are highly context dependent, and the planning and development of the models described by our interviewees was indeed subject to a range of contextual factors such as the availability of funds, the presence of one or more 'champions' to take the lead in development, the negotiation of local professional interests, and availability of trained workforce. The models which emerged were products of context, which shaped a process of local negotiations about the mechanisms which would best realise the policy ideal of shifting care into the community. [39] There was also often an element of serendipity in the process, with a chance coming together of key factors to create or impede change. [40]

Our findings resonate with a number of recognised theories of innovation and change management. We observed the described tension between centrally driven innovation and local adoption of 'good ideas',[11] and the paradox that the context, far from being a 'confounder' is integral to the implementation of complex innovation. [41] Our data exemplify the maxim that organisational change is subject to a range of variables which interact to influence outcomes. [11,40] The crucial significance of 'relative advantage', i.e. the need to identify models which offered advantages to all clinicians and managers who needed to be involved in development, was apparent as healthcare professionals impeded change that they perceived may be disadvantageous. [11] Champions are recognised as key determinants of organisational innovation,[11] echoing our interviewees accounts of how local professionals had successfully championed developments in their PCO. Such theories can provide insight into how the complex dynamics in some PCOs enable change to occur, whilst impeding change in others.

## Conclusion

Whilst some PCOs seemed able to overcome the challenges of organisational fluidity and financial constraints in order design and implement new services for people with long-term respiratory disease, the resulting services were largely directed at reducing admissions amongst the small number of people with complex needs. For many PCOs the barriers of financial deficit, organisational uncertainty, disengaged clinicians, and contradictory policies presented insurmountable barriers to the effective development of sustainable services. In other PCOs these barriers were being overcome and new models of care successfully developed, although their sustainability in the shifting organisational context at the time of the study was in question. Research should concentrate on understanding these complex dynamics in order to inform policy-makers, commissioners, health service managers and professionals of effective strategies to implement change.

## Abbreviations

Many of these explanations are based on, or reproduced with permission, from the NHS Jargon Buster: Version 2 (February 2008) Updated online at 

Acute Trust: A legal entity formed to provide health services in a secondary care setting.

Community Matron: When a patient has a number of long term conditions and complex needs, their care becomes more difficult for them to manage. Case Management is where a named coordinator, e.g. a Community Matron, actively manages care by offering continuity of care, coordination and a personalised care plan for vulnerable people most at risk.

COPD: Chronic Obstructive Pulmonary Disease

GP: General Practitioner Family doctor. Patients in the UK access healthcare through the GP practice with whom they are registered.

GPwSI: General Practitioners with a Special Interest. Practising GPs with a special expertise in (respiratory medicine) whose role often includes in service development as well as clinical care.

LDP: Local Delivery Plan. A 3 yr plan that every PCO prepares and agrees with its Strategic Health Authority (SHA) on how to invest its funds to meet its local and national targets, and improve services. It is a public document which provides an overview of PCO priorities, and how it intends to manage its resources.

LTC: Long-term conditions. Illnesses which lasts longer than a year, usually degenerative, causing limitations to one's physical, mental and/or social well-being. Symptoms may come and go, and usually there is no cure, but there are things that can be done to maintain or improve the person's quality of life and wellbeing. Long Term Conditions include Diabetes, COPD, Asthma, Arthritis, Epilepsy and Mental Health.

LTC pyramid: A pyramid with three levels of professional and self-care widely adopted as a model of service provision for people with long-term conditions. It is based on categorising care according to risk stratification.

NHS: National Health Service. The publicly funded healthcare system in England, Scotland, and Wales.

NSF: National Service Framework. These NHS documents set national standards for the provision of care for a range of disease areas.

PbR: Payment by Results. How secondary care providers in England are now paid. There is a national fixed tariff for emergency care, elective in-patients, day cases and outpatients bought by NHS commissioners. The important principle is that only work done and recorded using appropriate coding is paid for.

PCO: Primary Care Organisation. Freestanding statutory NHS bodies (Primary Care Trust in England; Local Health Boards in Wales) with responsibility for delivering healthcare and health improvements to their local areas. They commission or directly provide a range of community health services such as district nursing as part of their functions.

UK: United Kingdom

## Competing interests

The authors declare that they have no competing interests.

## Authors' contributions

HP initiated the idea for the study and led the development of the protocol and securing of funding with GH and AS. HP, GH, AT and AS contributed to study administration, data analysis, interpretation of results and writing of the paper. SH undertook the data analysis and wrote the original draft of the paper. All authors reviewed the final manuscript. HP and GH are study guarantors.

## Pre-publication history

The pre-publication history for this paper can be accessed here:



## Supplementary Material

Additional file 2 **Appendix 1.**  Screening interview topic guide. The topic guide used to structure the screening interviews. Click here for file

Additional file 1 **Table 1.** The PCOs: their demography of models of care, and role of interviewees Click here for file
