# Supplementary material for: Mind the gap between policy imperatives and service provision: a qualitative study of the process of respiratory service development in England and Wales
Source: BMC Health Serv Res. 2008 Dec 4;8:248. doi: 10.1186/1472-6963-8-248 (PMC2632662; doi:10.1186/1472-6963-8-248)
Supplement: Additional file 2 — Appendix 1. Screening interview topic guide. The topic guide used to structure the screening interviews. [file 1472-6963-8-248-S2.doc]

**Additional file 1. Screening interview topic guide**:

Schedule for the initial semi-structured screening interview with the person responsible for driving the reconfiguration of respiratory services in 30 PCOs.

**Background information**

Size of PCO:

Demographics: urban, semi-urban, rural, remote: areas of deprivation.

Workforce issues: GP vacancies, Consultant vacancies, nurse/physio availability.

Any specific local issues (unemployment levels, significant local employers competing for potential employees, local availability of training –university/colleges etc)

Plans for PCO reconfiguration: which are the proposed ‘partner PCOs’

**What are the key priorities for service redesign in your PCO?**

[Specific prompts: Key issues that affect service redesign, PCOs approach to the management of long-term diseases, How do respiratory services fit in with the overall strategy]

**Does your PCO have any plans to develop services for people with respiratory disease?**

**If yes:**

Please outline what service development is being considered or is already underway.

[Specific prompts: respiratory GPwSI, respiratory specialist nurse, Evercare/other managed care project, COPD Primary Care Collaborative, developing existing primary care/supporting GMS contract, secondary care outreach, Hospital at Home scheme, Providing specific services (spirometry, pulmonary rehabilitation, palliative care for COPD allergy)]

Why are the PCO considering these changes?

[Specific prompts: pressure on secondary care, primary care collaborative, strategic development of care for long-term conditions, pressure from a primary/secondary care respiratory champion, pressure from patient groups, SHA/national pressures]

[Any local information driving these decisions: referrals, waiting times, asthma and COPD admissions, prescribing costs]

[Any evidence informing these decisions: published literature, NatPact/BTS/NRTC/GPIAG/other resources, experience in neighbouring PCOs]

What are the priorities to be addressed by the reconfigured service?

[Specific prompts: reducing admissions, raising quality of primary care, reducing outpatient referrals, providing spirometry/pulmonary rehabilitation/palliative care for COPD/allergy services]

Who is responsible for driving changes (if any) in the provision of respiratory care, and/or other chronic disease areas?

[Specific prompts: PCO manager, primary/secondary care clinician]

[Other key players?]

What workforce changes will be needed to realise the planned development?

[Specific prompts: new appointments (GPwSI, specialist nurse/physic/other, healthcare assistants) new skills for existing staff (extending the skills of nurses/physios/healthcare assistants/other]

What training is planned for this reconfigured workforce? [Specific prompts: formal training (MSC, degree level, diploma level course, mentoring with local primary/secondary care clinicians, NRTC/RETC/other accredited training organisations/pharma sponsored training]

[Basis on which appropriate training was identified/chosen: formal needs analysis, clinician’s own preference, managerial decision, personal recommendation, official requirement]

[Accreditation/appraisal arrangements: local arrangements, following national guidance]

What are the barriers?

[Specific prompts: lack of suitable candidate(s) for new respiratory GPwSI/respiratory specialist nurse/physio/other posts, no funding for the new post, no funding to support training, opposition from primary/secondary, clinicians/PCO management/patients, competition with other priorities]

What sources of information and support have been accessed?

[Specific prompts: published literature, web-based advice e.g.NatPact/BTS etc, informal advice from colleagues]

What monitoring is planned?

[Specific prompts: COPD/asthma admissions/bed days, outpatient referrals, A&E attendances, PACT data, quality and outcome framework returns from the practices, referrals to new services]

What effect will the planned PCO reconfiguration have on these plans?

[Specific prompts: existing services/plans/respiratory champions in ‘partner’ PCOs, effect of uncertainties due to the reconfiguration]

Any other comments?

**If no:**

Please outline why reconfiguration of respiratory services is not a priority in your PCO.

[Specific prompts: existing primary/secondary service is very good, addressing the issues in other ways (what other models – eg generic CDM nurses) other priorities (what are these priorities and why?), no identified local need (what is this based on?), no local interest from clinicians]

[Factors that would change the priority attached to respiratory care: local data suggesting there was a problem, national/SHA directives, local interest/availability of specialists, identifiable funding stream]

What sources of information and support do you regularly access to help you develop services?

[Specific prompts: published literature, web-based advice e.g.NatPact, informal advice from colleagues]

What monitoring of respiratory services is routinely undertaken or planned?

[Specific prompts: COPD/asthma admissions/bed days, outpatient referrals, A&E attendances, PACT data, quality and outcome framework returns from the practices]

What effect will the planned PCO reconfiguration have on these plans?

[Specific prompts: existing services/plans/respiratory champions in ‘partner’ PCOs, effect of uncertainties due to the reconfiguration]

Any other comments?

**Thank-you for helping with our research.**

The information you have given us will help us understand how respiratory care is being developed around the country. In the next phase of this project we will be recruiting 6 PCOs who are planning different models of care to take part in an in-depth case-study over the next year. If we think that your PCO would be a particularly useful example for our study, please may we approach you again to see if you would be interested?
