# Supplementary material for: Mind the gap between policy imperatives and service provision: a qualitative study of the process of respiratory service development in England and Wales
Source: BMC Health Serv Res. 2008 Dec 4;8:248. doi: 10.1186/1472-6963-8-248 (PMC2632662; doi:10.1186/1472-6963-8-248)
Supplement: Additional file 1 — Table 1. The PCOs: their demography of models of care, and role of interviewees [file 1472-6963-8-248-S1.doc]

**Table 1. The PCOs: their demography of models of care, and role of interviewees**

| **PCO** | **Population (to nearest 50,000)** | **Deprivation,**  **Any special features** | **Financial status** | **Role of interviewee** | **Model of respiratory care** |
| --- | --- | --- | --- | --- | --- |
| 1 | 250,000.  Urban, | Some areas of deprivation | In deficit | Manager  (Nursing) | Respiratory specialist nurses, Community Matrons,  GPwSI referral service |
| 2 | 200,000.  Rural/coastal, | Some areas of deprivation Elderly population | Unknown | Manager  (Service development) | Community matron |
| 3 | 150,000.  Urban, | Some areas of deprivation | In balance | Commissioner | GPwSI and specialist nurse,  Acute trust respiratory nurses, Community Matrons, |
| 4 | 150,000.  Urban, | Some areas of deprivation | In deficit | Manager (Governance) | Respiratory specialist nurses,  Acute trust respiratory nurses |
| 5 | 150,000.  Urban/rural, | Mostly affluent | In deficit | Manager  (Service development) | No respiratory service |
| 6 | 300,000.  Small city, | Some areas of deprivation | In balance | Commissioner | Respiratory specialist nurses, |
| 7 | 100,000. Urban/rural/remote | Mostly affluent | In balance | Manager  (Nursing) | Respiratory specialist nurses and Community Matrons |
| 8 | 150,000.  Urban/rural, | Mostly affluent | In deficit | Manager  (Service development) | Respiratory specialist nurses,  Interested GP |
| 9 | 200,000.  Urban/rural, | Some areas of deprivation | In deficit | Manager  (Service development) | Acute trust respiratory nurses, Pulmonary rehabilitation,  Practice leads |
| 10 | 200,000.  Inner city, | High levels of deprivation | Unknown | Manager  (Nursing) | Respiratory specialist nurses, Community Matrons,  Pulmonary rehabilitation, |
| 11 | 200,000.  Urban/rural/remote | Some areas of deprivation | Unknown | Manager  (Service development) | Respiratory specialist nurses,  Practice leads |
| 12 | 150,000.  Rural, | Some areas of deprivation | In deficit | Manager  (Service development) | Acute trust respiratory nurses and community consultant,  Pulmonary rehabilitation |
| 13 | 250,000.  Urban, | Some areas of deprivation | In balance | Commissioner | Acute trust respiratory nurses,  Urgent care centre with community consultant |
| 14 | 100,000.  Urban/rural, | Relatively affluent | In deficit | Respiratory GPwSI | GPwSI and specialist nurse, |
| 15 | 200,000.  Rural, | Some areas of deprivation | In deficit | Manager  (Service development) | Respiratory specialist nurses, Physiotherapist, Community matrons, Nurse educator in primary care, |
| 16 | 150,000.  Urban/rural, | Some areas of deprivation | In deficit | Respiratory GPwSI (not in post) | Respiratory specialist nurses,  Potential GPwSI |
| 17 | 300,000.  Rural/coastal, | Some areas of deprivation  Elderly population | In deficit | Respiratory GPwSI | GPwSI, Respiratory specialist nurses, Physiotherapist,  Nurse educator in primary care |
| 18 | 150,000.  Urban/rural, | Some areas of deprivation | In balance | Commissioner | Respiratory specialist nurses, Community Matrons |
| 19 | 200,000.  Urban/rural. | Relatively affluent | In deficit | Manager  (Service development) | Acute trust respiratory nurses, Pulmonary rehabilitation |
| 20 | 150,600.  Rural/remote. | High levels of deprivation,  Ex-mining community | In deficit | Manager  (Nursing) | Respiratory specialist nurse |
| 21 | 100,000.  Inner city, | High levels of deprivation, | In balance | Manager  (Service development) | Respiratory specialist nurses, Community Matrons, Pulmonary rehabilitation, Consultant and nurse support for primary care, |
| 22 | 250,000.  Inner city, | Some areas of deprivation | In balance | Manager  (Service development) | Acute trust respiratory nurses, Consultant outreach clinics,  Pulmonary rehabilitation |
| 23 | 200,000.  Suburban, | Mixed affluence/deprivation | In balance | Manager  (Nursing) | Respiratory specialist nurses, Telemedicine |
| 24 | 110,000.  Urban. | Mixed affluence/deprivation | In balance | Manager  (Nursing) | Respiratory specialist nurse and physiotherapist,  Community Matrons |
| 25 | 300,000.  Rural. | Some areas of deprivation | In deficit | Commissioner | Respiratory specialist nurse and physiotherapist,  Community Matrons |
| 26 | 100,000.  Rural, | High levels of deprivation  Ex-mining community, | In balance | Manager  (Nursing) | Community Matrons |
| 27 | 100,000.  Rural. | Mostly affluent,  Elderly population | In balance | Manager  (Service development) | Upskilling primary care,  Pulmonary rehabilitation |
| 28 | 150,000.  Urban/rural. | Mixed affluence/deprivation | In deficit | Manager (Nursing and social services) | Acute trust respiratory nurses |
| 29 | 150,000.  Urban / coastal. | High levels of deprivation  Ex-mining community | In balance | Manager  (Service development) | Acute trust respiratory nurses, Physiotherapist.  Consultant outreach clinics |
| 30 | 250,000.  Urban, | High levels of deprivation | In deficit | Commissioner | Acute trust respiratory nurses, Pulmonary rehabilitation,  GPwSI |
